# One Soft Step: Bio-Inspired Artificial Muscle Mechanisms for Space Applications

**DOI:** 10.3389/frobt.2021.792831

**Published:** 2022-01-06

**Authors:** Joseph Ashby, Samuel Rosset, E.-F. Markus Henke, Iain A. Anderson

**Affiliations:** ^1^ Biomimetics Laboratory, Auckland Bioengineering Institute, University of Auckland, Auckland, New Zealand; ^2^ Institute for Semiconductors and Microsystems, TU Dresden, Dresden, Germany; ^3^ StretchSense (Sensor Holdings Ltd.), Auckland, New Zealand; ^4^ Dresden Center of Intelligent Materials (DCIM), TU Dresden, Dresden, Germany; ^5^ PowerOn Ltd., Auckland, New Zealand

**Keywords:** dielectric elastomer actuator, space robot, artificial muscle, inflatable actuators, soft robotics, smart material, electroactive polymer

## Abstract

Soft robots, devices with deformable bodies and powered by soft actuators, may fill a hitherto unexplored niche in outer space. All space-bound payloads are heavily limited in terms of mass and volume, due to the cost of launch and the size of spacecraft. Being constructed from stretchable materials allows many possibilities for compacting soft robots for launch and later deploying into a much larger volume, through folding, rolling, and inflation. This morphability can also be beneficial for adapting to operation in different environments, providing versatility, and robustness. To be truly soft, a robot must be powered by soft actuators. Dielectric elastomer transducers (DETs) offer many advantages as artificial muscles. They are lightweight, have a high work density, and are capable of artificial proprioception. Taking inspiration from nature, in particular the starfish podia, we present here bio-inspired inflatable DET actuators powering low-mass robots capable of performing complex motion that can be compacted to a fraction of their operating size.

## Introduction

This paper presents bio-inspired inflatable actuators for space applications based on the starfish foot. The actuators combine mass reduction, deployable structures and polymer-based smart materials as methods for decreasing spacecraft mass while retaining functionality.

The cost of launching a kilogram of mass into Low Earth Orbit on the NASA space shuttle was in excess of $54,000 USD (Pielke, 1994). Though this has decreased twenty-fold with the advent of commercial launch platforms (Jones, 2018), spacecraft are still very constrained in terms of both mass and volume. One method for getting around these limitations is to launch many small structures and join them together once they are in orbit; this is how the International Space Station (ISS) was constructed. Another option is to design systems such that they can be stowed in a small volume, and then deployed at a later time. The ways this can be achieved include simple systems with springs, complex origami structures (Pehrson et al., 2020), and inflatable structures (Freeland et al., 1998; Tarazaga et al., 2007; Chandra et al., 2018).

An alternative approach is to reduce the mass of the spacecraft by replacing traditional technology with more modern, lighter counterparts. Robotic actuators in space are almost always some form of DC electric motor, used due to their long history of reliable performance (Allegranza et al., 2014). However, these motors are often heavy, and usually require the addition of gearing and/or flexures to function, adding yet more mass. In recent years there has been a growing interest in smart material alternatives. Among these emerging technologies are electroactive polymers, soft materials which deform in response to an applied electric field. This technology is further separated into materials which use the motion of ions to bring about deformation (ionic electroactive polymers), and those which are actuated by the coulomb forces produced by the electric field itself (dielectric Elastomer Transducers (DETs)) (Brochu and Pei, 2010).

Our project explores the potential for inflatable soft robots in space powered by smart skins embedded with DETs. Once the concepts have been verified it is our intention to improve the Technology Readiness Level of inflatable DET systems by testing them in simulated space conditions, taking them a step closer to practical use in space.

Unlike traditional robotics, the design and control of inflatable DETs is a complex issue. So what we, and many other researchers have done, is look to nature for inspiration. The earth is full of soft-bodied fauna that provide unique ways for interacting with multiple environments. We have chosen the starfish podium (also known as a tube foot) as the system we wish to emulate. Starfish are capable of traversing a huge variety of terrain both above and below the water, in any orientation, all using arrays of these legs. In space this would translate to an incredibly versatile locomotory system able to operate in zero gravity and on many of the different surfaces encountered by spacecraft, such as: the smooth, rigid exterior of larger man-made satellites; the rocky, dusty surface of an asteroid; or even extra-terrestrial fluid bodies like those recently discovered on Mars (Lauro et al., 2020).

**FIGURE 1:**
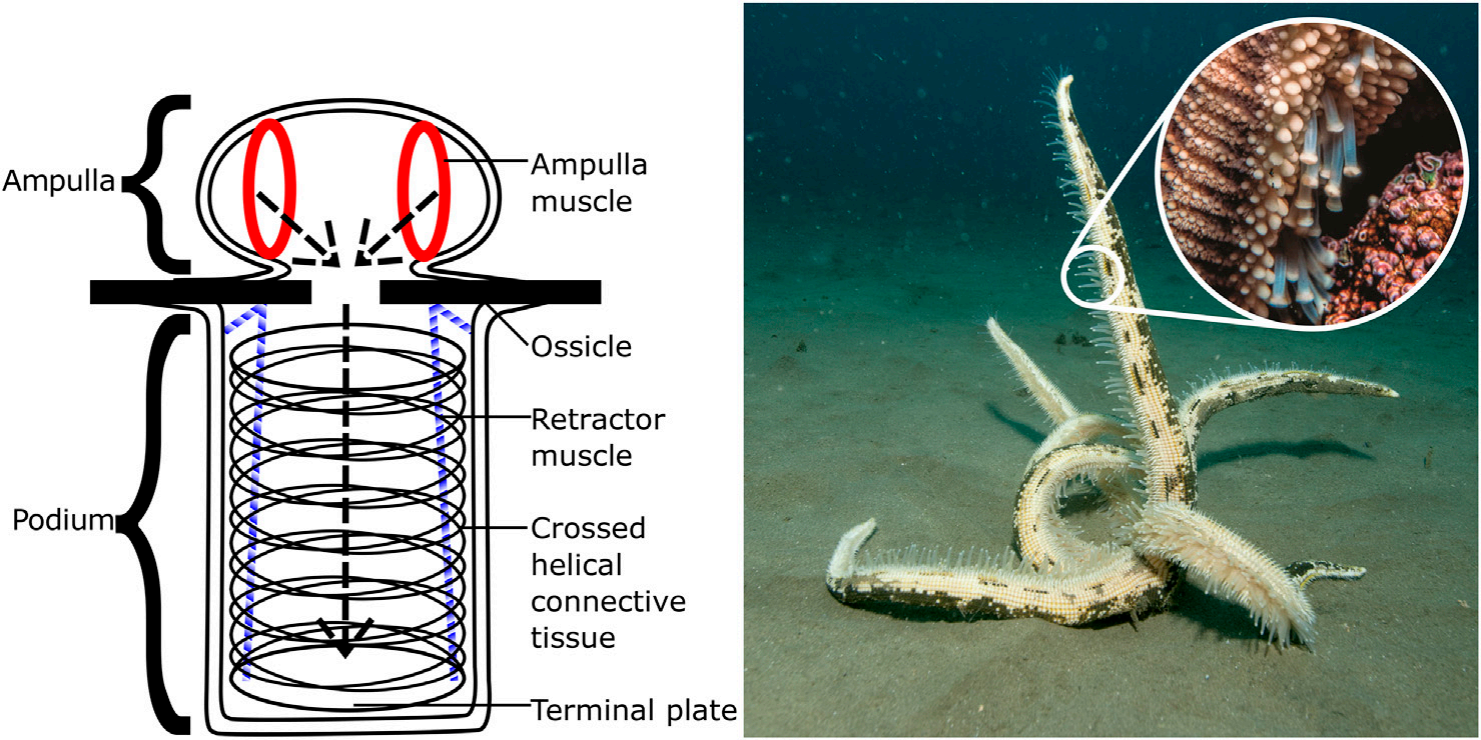
Left: Basic structure of the podium (tube foot) of the common starfish, *Asterias Rubens*, showing the muscles used for contracting and deforming the ampulla and podium. Right: A starfish righting itself on the sea floor, with close-up of the leg showing many podia in actuation [photographs by Iain Anderson].

In the starfish, each “foot” is separated into two sections (see Figure 1): the podium itself on the exterior of the body, its structure maintained by rings of connective tissue and manipulated by longitudinal muscles; and the ampulla, an internal sac capable of compressing itself. This compression forces water into the podium, causing it to expand, and likewise when the ampulla muscles relax the water is forced back into the ampulla and the podium shrinks. In combination with the orientation control provided by the podium muscles, each foot is capable of being extended, retracted, and bent in multiple directions.

The starfish podium is filled with fluid from their water vascular system, and, while ideally suited to work in the undersea environment, due to the aforementioned mass restrictions we turn to the use of gas as an inflation medium for operation in the atmosphere and beyond.

### Inflatable Space Structures

Inflatable structures are not new to space, in 1960 and 1964 NASA launched a pair of large (>30 m diameter) aluminized mylar balloons into orbit, Echo 1 and 2 (Hansen, 1995). Echo 1 was successfully used as a passive communications satellite for several months. Recent interest in inflatable structures has increased due to the success of the Bigelow Expandable Activity Module (BEAM), a storage module for the ISS which completed its initial 2-years mission and has remained docked for extended usage (Valle and Wells, 2018). The majority of inflatables designed for space are structural, for habitation, storage, or large communication structures (antennae, reflectors). These designs are mostly intended to be inflated and rigidised to form inflexible passive structures. This rigidisation can be achieved in multiple ways, using hardening resins (UV, temperature, and etc) or strain-rigidising metal-polymer laminates (Schenk et al., 2014). However in this work we investigate the potential for inflatable robotic systems that are active and flexible.

The problems with using gas in space are primarily those of control, and storage. An early attempt to launch Echo 1 ended in disaster as small amounts of residual air deliberately left inside the balloons, intended to inflate it, expanded far more rapidly than planned, tearing the skin to shreds. The late 90s saw a similar incident with the L’Garde Inflatable Antenna Experiment, a test wherein a 14 m parabolic reflector supported by an inflatable torus and 3 inflatable struts was deployed from a satellite on-orbit. Small amounts of residual air caused rapid, uncontrolled inflation of the torus, and uneven deployment of the struts (Freeland et al., 1997). These experiments show that using residual gas for inflation is effective but dangerous; better methods allow for the controlled release of gas into the structure at a slower rate. This can be done with traditional compressed gas containers, as was the case for BEAM, but only for large scale structures. A more scalable method is the use of sublimating solids, the eventual method used by the Echo project. Although manual inflation was the only method used in the experiments described in this paper, alternatives will be the focus of a future publication.

Soft robots show great potential for space applications (Lin et al., 2013), and DETs could be an integral part of their development. To be completely soft, these robots would require soft actuators, and current inflatable designs typically use pneumatic actuators (for example the McKibben actuator or derivatives) powered by large tethered compressors. These compressors have considerable bulk, and make the use of these robots in space less practical. Our approach to this problem is to replace the pneumatic actuation with electrical. By integrating DETs into the skin of a robot we can selectively deform different sections to produce a desired motion. This allows for the production of untethered, lightweight inflatable robots.

### DETs in Space

**FIGURE 2:**
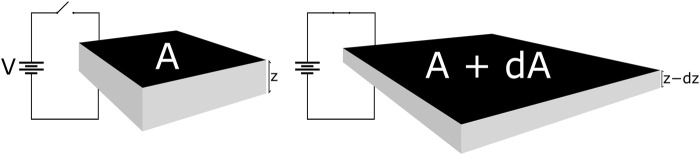
Operating principle of a Dielectric Elastomer Actuator. A is the area of the electrodes and z is the thickness of the dielectric layer, prior to actuation. dA represents the increase in area and dz is the decrease in thickness, after actuating. Applied voltage V is typically on the order of 3–5 kV for a membrane thickness of 0.1 mm.

The basic structure and function of a DET actuator (DEA) is shown in Figure 2. In its simplest form, it is a sheet of elastomer with compliant electrodes on either side to form a soft capacitor. These stretchy electrodes can be produced from a wide variety of materials (Rosset and Shea, 2013). Carbon based electrodes are the most common in literature, either as a loose powder, suspended in a grease, or as part of a conductive polymer matrix as in our case. When a voltage is applied, the subsequent Maxwell pressure causes the area to expand and the thickness to decrease (Pelrine, 2000). DEAs have been used to power a huge variety of different devices since their inception. When placed in antagonistic arrangements they can mimic biological structures like the human eyeball (Liu et al., 2008), jaw (Wang and Zhu, 2016), limbs (Kovacs et al., 2007) or other locomotory systems (Sun et al., 2016; Cao et al., 2018). Of particular inspiration for us were the multi-degrees-of-freedom spring roll actuators used by Pei *et al.* used to create a hexapod robot (Pei et al., 2004).

On Earth, DETs have proven to be excellent candidates for actuation, strain-sensing (the capacitance of DETs increases with strain), and energy generation (Anderson et al., 2012). For example, as strain sensors they have been used to monitor the human body in motion-capture gloves (StretchSense, 2020) and diver body kinematics with gesture recognition (Walker and Anderson, 2017; Nad et al., 2019). DETs have also been produced that can sense compression, and shear forces which have potential applications in fields as varied as health care and agriculture (Mahmoudinezhad et al., 2020; Kim et al., 2013).

For space, DETs have undergone testing as sensors for monitoring the structural health of inflatable structures (Litteken, 2017) due to their excellent strain-sensing properties, ability to undergo large deformations, and their ease of integration into fabric. There have also been some attempts to produce space actuators using DEAs including a robotic arm (Branz and Francesconi, 2016) and a satellite gripper (Araromi et al., 2015). These actuators used rigid structures to provide the agonist to the DEA antagonist. Where our actuators differ is the use of inflation, allowing them to be more tightly compacted prior to deployment and making them capable of forming entirely different structures to those comprised of rigid materials.

DET grippers have also been built which incorporate electro-adhesion, utilising electrical fringe fields produced by interdigitated electrodes to generate attractive forces in nearby objects or surfaces (Shintake et al., 2016). Though the forces produced by these electro-adhesive pads are small, this is not a major disadvantage when operating in a zero gravity environment. One area in which this adhesive mechanism excels where others do not is controllable adhesion to smooth, non-magnetic surfaces (or surfaces which should not be magnetised, e. g due to sensitive electronics), such as the exterior of man-made satellites. Electro-adhesive pads have been used in combination sensor/gripper configurations such as that published by Xiang *et al.* which are integrated into inflated polymer shells similary to those used in this work and use the softness of the structure to conform to, and then grip, lightweight objects of varying surface curvature (Xiang et al., 2019). Electro-adhesive pads are an excellent addition to an actuator, but here we will primarily focus on the mechanical movement.

In this paper we focus on two designs in particular. The first design is a segmented ball-shaped actuator with three degrees of freedom, equivalent to a universal joint coupled to a linear actuator. The second is a wavy hemi-spherocylidrical design, only capable of moving in two dimensions. However it is capable of acting as a linear conveyor in the same way as a line of multiple instances of the first design. This was derived from the first design to accentuate one potential use (linear conveyance/locomotion) while sacrificing the ability to move in more than one plane.

## Methods

This methodology encompasses the design, modelling, and fabrication of our actuators.

### Inflatable Robot Design

Our inflatable structures with integrated DEAs, start with flat membranes inflated from one side. The basic design was an inflated dome incorporating segmented carbon electrodes capable of tilting the dome in any direction called MIDA (multi-directional inflatable dome actuator). Further iterations we have built include the addition of an electro-adhesive end effector (to allow it to grip nearby objects or surfaces) (Ashby et al., 2019) and miniaturisation to demonstrate more advanced production techniques (inkjet printing) (Ashby, 2019).

**FIGURE 3:**
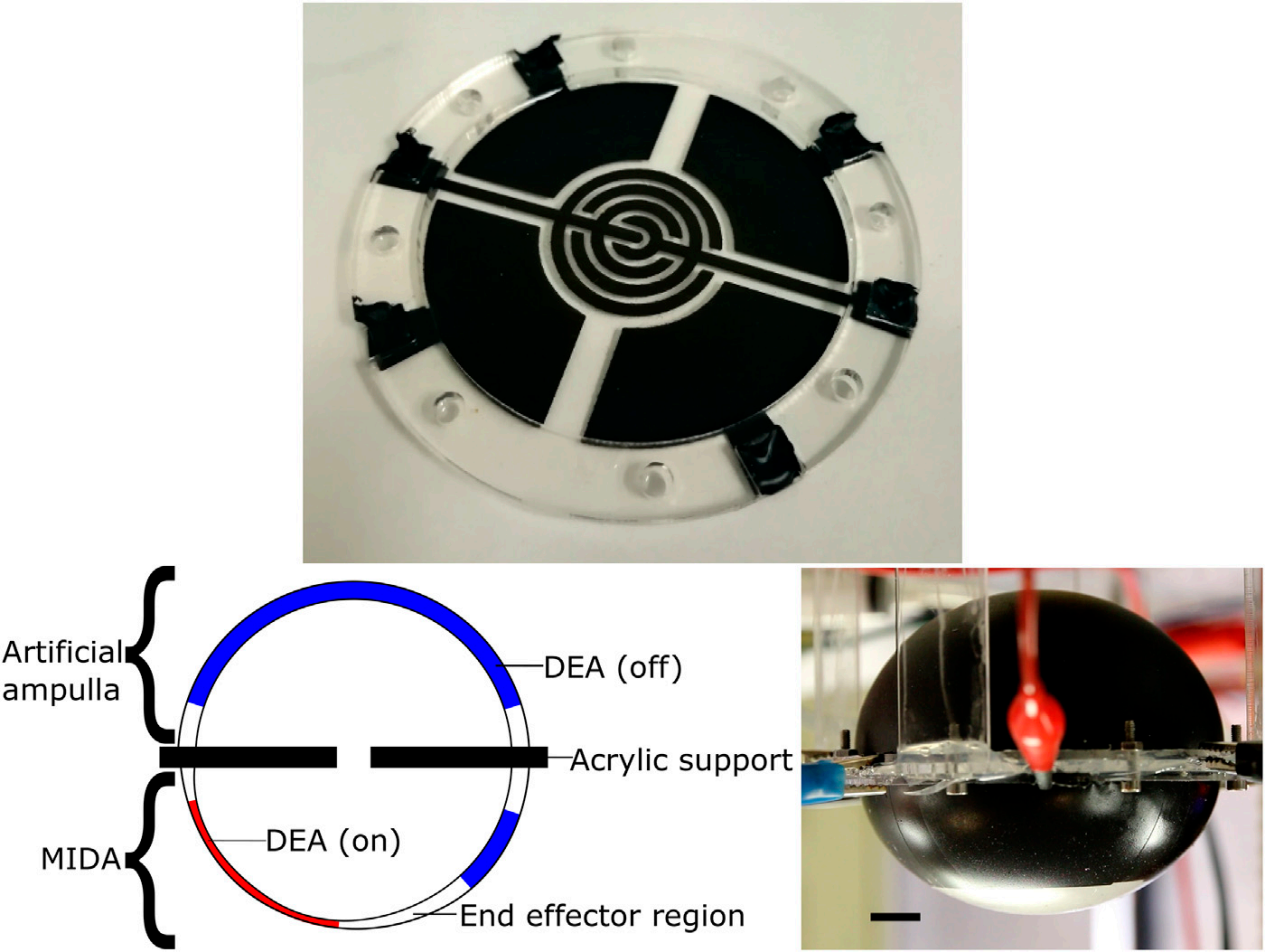
Top: The top electrodes of a MIDA model, showing the 4 segmented actuators and an electro-adhesive end effector. Left: The structure of the MIDA artificial tube foot actuator, inflated and undergoing actuation. Right: The latest prototype MIDA, inverted. Black line is 1 cm in length. See Supplemental Materials for a video of the actuator in motion.

**FIGURE 4:**
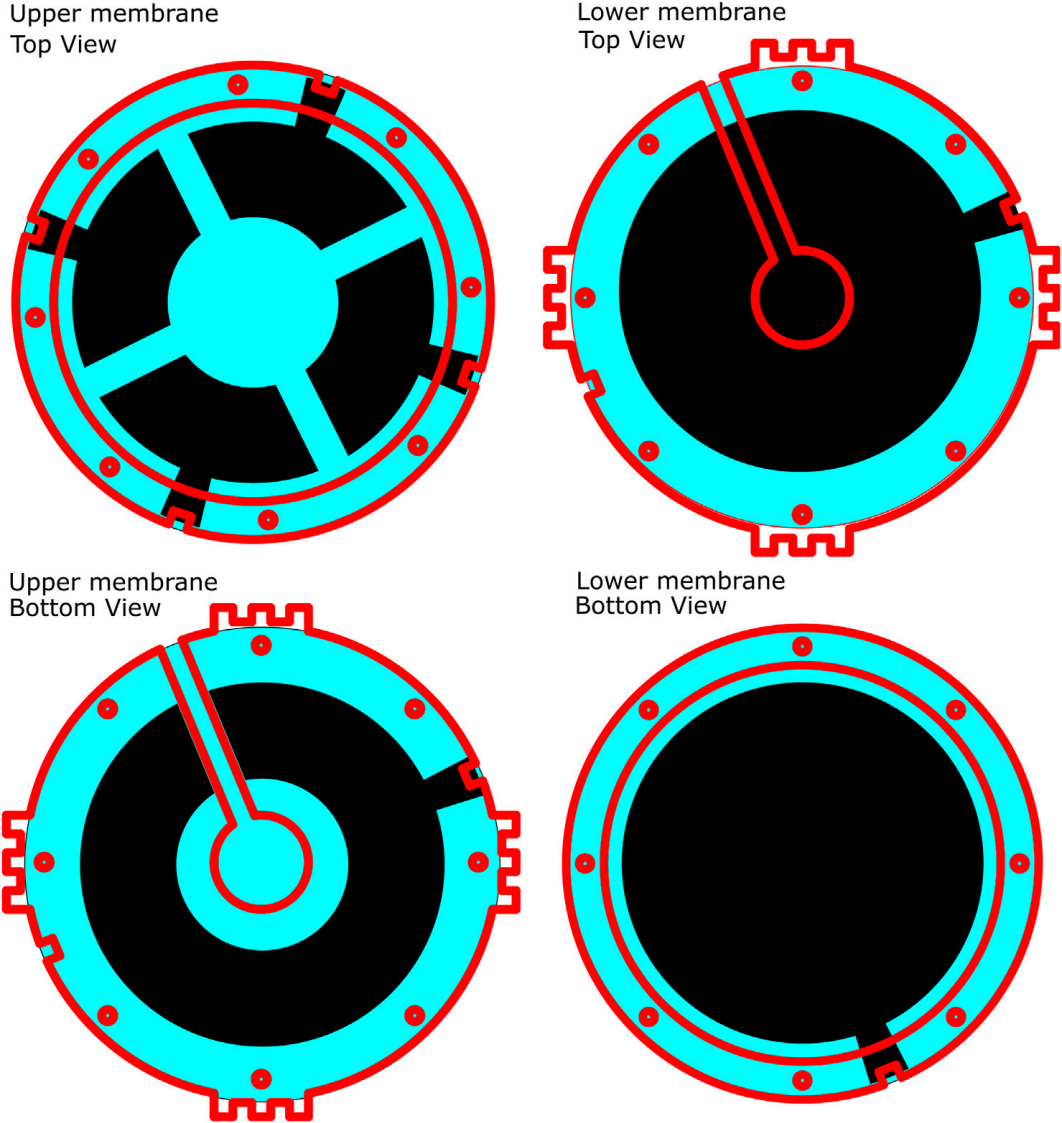
Structure of the MIDA test setup in the deflated state, red lines indicate the position of the edges of acrylic support frames. Top Left: Top view, upper membrane. Top Right: Top view, lower membrane. Bottom Left: Bottom view, upper membrane. Bottom Right: Bottom view, lower membrane. Small holes show bolt placement, air channel visible on Top Right and Bottom Left.

**FIGURE 5:**
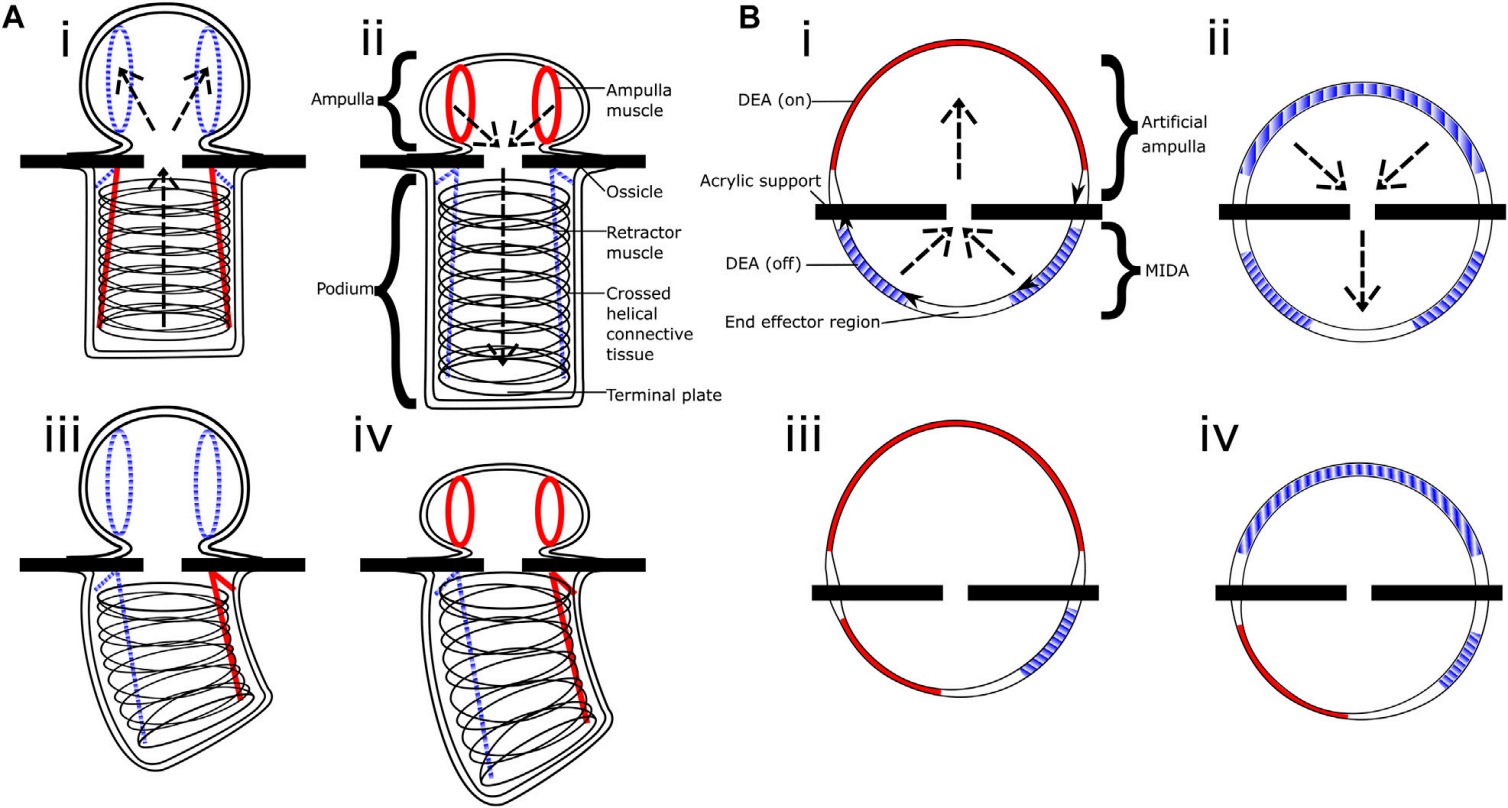
Comparative diagram. **(Ai–iv)** shows the basic structure and motions of the tube foot of *Asterias Rubens* the common starfish, whilst **(Bi–iv)** shows the equivalent in our inflated actuator. Sections in red/solid are active muscles and DEAs, blue/striped are inactive, you can see the operation is inverted between the contracting biological muscle and our expanding artificial muscles. The operations of the ampulla and artificial ampulla are shown in figures **(Ai–ii,Bi–ii)** respectively, the arrows indicate the water/air flow.

The latest version of MIDA took inspiration from the starfish podia, see Figure 3. As well as the dome with segmented electrodes it incorporated a second side, this one comprising a single large actuator, an artificial analogue for the biological ampulla (see Figure 4). When actuated this dome expands, causing the original MIDA side to contract, as illustrated in Figure 5. This gives the system a fully 3D range of movement. Figure 5 shows the parallels between the motion of the starfish podium and that of our actuator. A key difference is the operation of the “muscles;” actuation of the DETs causes an expansion as opposed to the contracting biological muscle.

**FIGURE 6:**
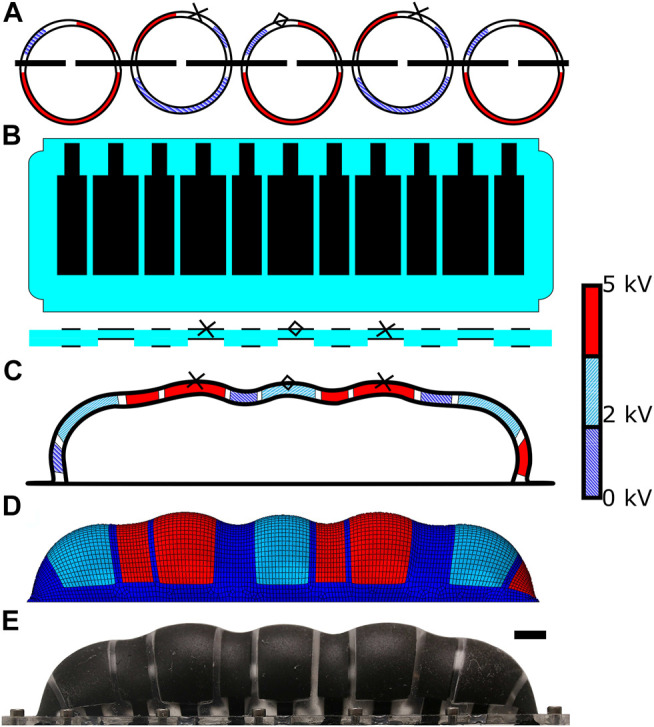
PILA, the phased inflatable locomotory actuator. **(A)**—a series of MIDA actuators in a line, the structure PILA is intended to emulate. The crosses and diamonds on **(A–C)** show points that travel along equivalent paths (see Figure 14). **(B)**—PILA in a deflated state, showing the relative size and position of the electrodes and a representative cross section showing the regions of different thickness (not to scale). **(C)**—the basic layout of PILA when inflated showing a cross section through the central axis, DETs are highlighted in various colours to show level of actuation at this point in the cycle. **(D)**—Finite element model of PILA undergoing a standard walking cycle using 3 kV peak to peak square wave (with DC offset 3.5 kV) actuation for the “feet” electrodes and 5 kV peak to peak (with 2.5 kV DC offset) sine wave actuation for the displacement electrodes. **(E)**—Physical prototype PILA. Black line is 1 cm in length. See Supplemental Materials for a video of the actuator in motion.

**FIGURE 7:**
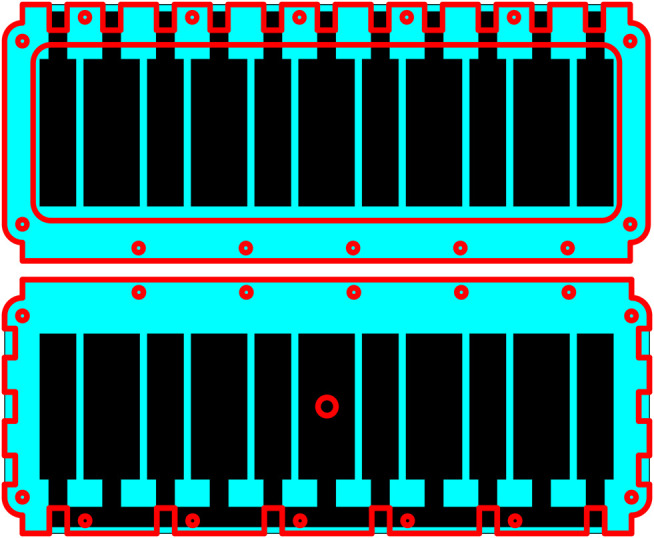
Structure of the PILA test setup, red lines indicate the position of the edges of acrylic support frames. Top: Top view. Bottom: Bottom view. Small holes show bolt placement, large central hole shows air tube placement.

**FIGURE 8:**
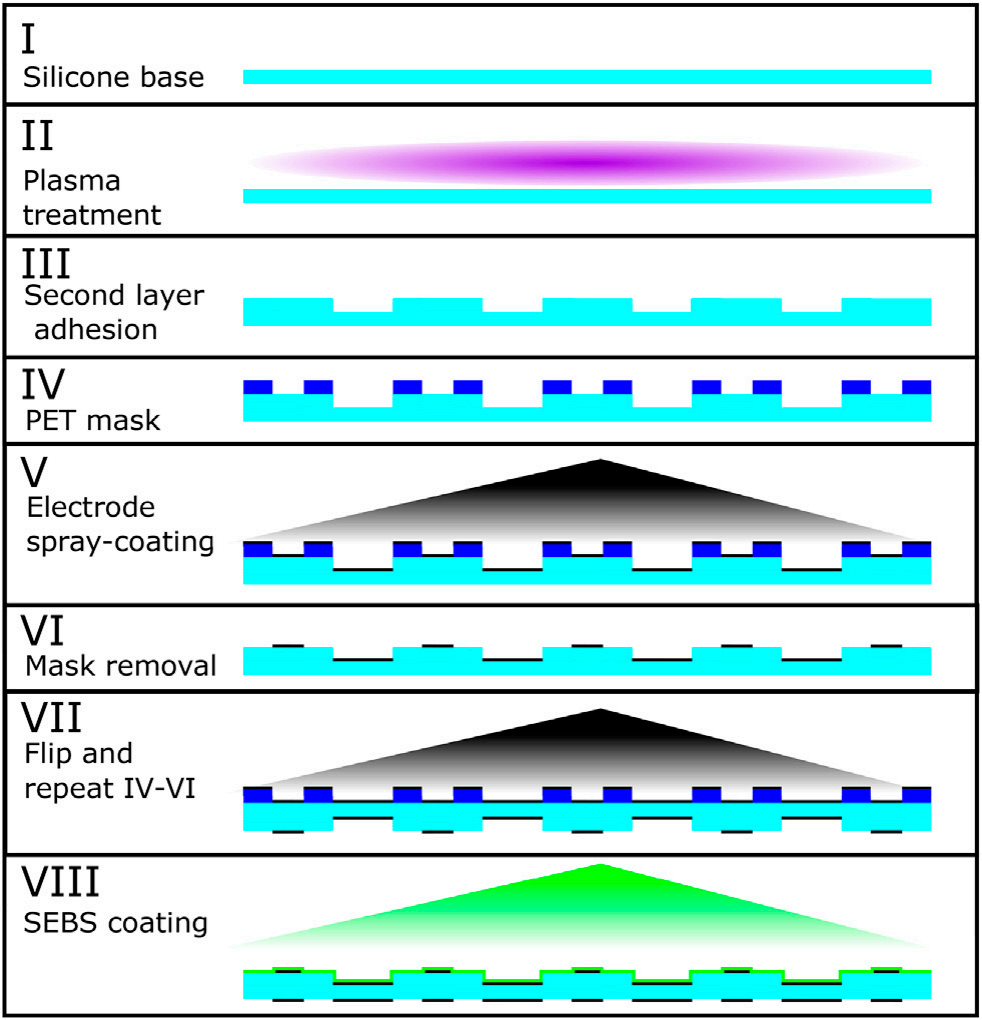
Production of the PILA actuator. Starting at stage I with the silicone base layer (Wacker Elastosil^®^2030), this is plasma treated (II) to activate the surface for bonding with a similarly tre’ated second layer to produce the different thickness sections (III). A PET mask is used (IV) to selectively spray-coat the conductive electrode mixture onto the active areas (V). This process is then repeated on the other side for the ground electrodes (VII). Finally a thin layer of SEBS-g-MA (maleic anhydride grafted styrene-ethylene-butylene-styrene) co-block polymer is applied to reduce gas permeability.

In the starfish, a single podium cannot effect much force; many podia work in tandem to produce enough force to move the starfish. Rather than create an array of individual MIDA actuators, we produced the same functionality in a single model. To that end, we designed our Phased Inflatable Locomotory Actuator (PILA, see Figure 6). PILA is a hemi-spherocylinderical inflated conveyor system which uses an array of DETs, to provide similar functionality to multiple MIDA actuators using a single-sided structure (see Figure 7). In MIDA a simple 100 µm thick silicone membrane is used, in PILA it is instead two 50 µm thick silicone sheets bonded together using plasma treatment (see Figure 8), with one having patterned holes so as to produce regions of half thickness, see Figure 6B. When inflated, this becomes a naturally wavy structure, see Figure 6C. The “peaks” are the thinner and wider sections that produce the out-of-plane motion, similar to the ampulla of MIDA and the starfish podia, the “trough” actuators provide the horizontal displacement of the “feet” necessary for the walking motion. This walking motion is an approximation of a “metachronal wave” that is seen in nature not only for the motion of starfish legs but also some terrestrial multi-legged animals such as centipedes. In contrast to the looping gait of leeches and caterpillars (and robots based on them such as (Guo et al., 2020)) it is a more dynamic gait, capable of relatively fast locomotion.

The maximum theoretical speed of either walking or conveying a load (assuming perfect operation and ignoring weight in the case of walking), is simply the horizontal displacement of one foot during a cycle multiplied by twice the frequency (twice because one foot will pass the load immediately to the next at the mid-point of the cycle). At higher frequencies we see a decrease in conveyor speed as the DEAs do not fully actuate, and the length of each stride decreasing is not compensated for by the faster steps. This difficulty in predicting the motion can be understood through computational modelling.

### Modelling

DEAs can be modelled analytically for very simple geometries. For small actuation of an ideal, flat elastomer of constant volume (*Adz* + *zdA* = 0), the Maxwell pressure can be written as:

$$P=\epsilon_{r}\epsilon_{0}E^{2}$$ (1)

Where *ϵ*_0_ is the permittivity of free space, *ϵ*_*r*_ the relative permittivity of the dielectric, and E the applied electric field (Suo, 2010).

Using this we can estimate the induced strain in the *z* direction:

$$S_{x}=\frac{P}{Y}=-\frac{\epsilon_{r}\epsilon_{0}E^{2}}{Y}=-\frac{\epsilon_{r}\epsilon_{0}V^{2}}{Yz^{2}}$$ (2)

Here we have used $\frac{V}{z}$ to define the electric field *E* across the actuator and *Y* as the Young’s modulus of the dielectric, which is assumed to be constant for small deformations. This very simplified model assumes a perfectly elastic material, whereas in reality most DETs must be modelled as hyper-elastic systems (Suo, 2010).

For predicting large deformation of materials with a non-linear stress-strain curve, different models of varying complexity have been developed. One of the first, the Neo-Hookean model for hyper-elastic materials undergoing large deformation (proposed in 1948) is based on the thermodynamics of cross-linked polymer chains. This model is simple however, and tends to be accurate only for low strains. Polymer chains have a maximum strain before they reach their limit whereupon a drastic increase in the Young’s modulus will occur, this effect is not seen in the Neo-Hookean model, but is in more recently developed models (Suo, 2010). Some modern models used for predicting the strains of hyper-elastic systems include the Arruda-Boyce, Ogden, Mooney-Rivlin, Yeoh, and Gent models (Rosset et al., 2014; Gu et al., 2017; White et al., 2014; Vertechy et al., 2012; Lampani, 2000; Wissler and Mazza, 2005b,a, 2007).

Analytical models however are not feasible for more complex structures, and so the implementation of Finite Element Modelling (FEM) as a numerical method is a useful tool for the design and prediction of DETs. The model used in this work is a based on that developed by O’Brien *et al.* in their 2009 paper (O’Brien et al., 2009), using ABAQUS FEA and custom subroutines UHYPER and USDFLD to simulate hyper-elastic behaviour and the applied voltage respectively. The primary difference is the form of the Strain Energy Function. For this the Yeoh model was used instead of the modified Arruda-Boyce as it has previously demonstrated excellent conformity to experimental results for Elastosil 2030 (Henke et al., 2017). The material constants were those identified by Kuhring *et al.* in their 2015 paper (Kuhring et al., 2015). The elements were M3D8R, 8-node quadratic membrane elements with reduced integration, which were generated in the deflated configuration. Inflation was simulated through application of a uniform pressure to the inside of the membrane surface prior to actuation. Other parameters are given in Table 1.

**TABLE 1:** Parameters used for FEM simulation of DEAs. Yeoh parameters were taken from (Kuhring et al., 2015), dielectric permittivity taken from manufacturer specifications (Wacker Chemie AG, 2020).

FEM parameter	Value
ABAQUS element type	M3D8R
Initial Thickness	50/100 µm
Yeoh material properties *C*_10_, *C*_20_, *C*_30_	181.1, −159.8, 6.29 kPa
Relative permittivity	2.8

**FIGURE 9:**
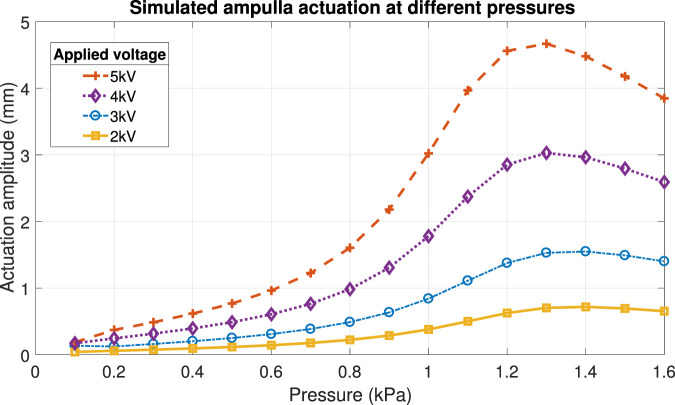
Finite element modelling of the actuation amplitude of the MIDA ampulla at varying pressures and applied voltages.

This model was used to optimise our design, by investigating the effect of changing parameters like the geometry of the electrodes (and the thin sections in PILA) without having to produce multiple physical prototypes. Another important factor in actuation is the level of inflation, an increase in actuation (assuming a constant voltage) is seen at higher pre-strains due to the thinning of the silicone and thus a higher electric field, but is counteracted by the strain-stiffening effect at higher inflations. The optimum inflation level can be found experimentally or by running simulations at different pressures and monitoring the level of actuation. See Figure 9 wherein the actuation (peak to peak displacement of the centre) of the ampulla was simulated at increasing pressures and voltage. The voltage used was a sinusoidal wave with a DC offset equal to half of the peak to peak amplitude, such that the voltage oscillated from 0 V to the maximum (2–5 kV). A local maximum can be observed for all curves at 1.3–1.4 kPa.

### Production Methods

The production of the experimental prototypes is outlined in Figure 8, which shows the stages involved in producing the active membrane of a PILA actuator. We started with a pre-made silicone membrane (Wacker Elastosil^®^2030, 50 µmfor PILA, 100 µm for MIDA) which was supplied on a roll with PET backing. From this we laser-cut a membrane of the desired shape. In the case of PILA, a second membrane was also cut, with gaps where thinner sections were desired. These membranes were then treated with a hand-held laboratory corona treater (Electro-Technic model BD-20AC) and pressed together to form the final bonded structure, this method was first published by Haubert *et al.* (Haubert et al., 2006). The treater was affixed to an acrylic holder which was set to maintain a 5 mm distance from the membrane at a 90°angle. Each membrane was treated for 45 s.

The electrodes used were a silicone-carbon black composite (Smooth-On EcoFlex™5 and Vulkan XC-72R respectively, 4.76% carbon black by weight after curing), which was thinned with heptane to reduce the viscosity. This was applied through a PET mask using an airbrush with a 0.2 mm nozzle (Harder & Steenbeck Ultra), at a pressure of 200 kPa. The final electrodes have a sheet resistance of around 100 k℧.

The membrane was affixed to a laser-cut acrylic support frame using an RTV silicone (Dow Corning 734) after stage VI in Figure 8 to allow the removal of the backing layer before the application of the second electrode layer. This frame was later attached to an acrylic support structure after stage VII which incorporates channels for inflation and cutaways to allow access to the electrode layers (see Figures 4, 7).

The silicone membrane used was gas-permeable, as is typical for PDMS compounds, albeit only to a small degree. However, maintaining a particular level of inflation over a long period was vital to the function of our actuators. To that end we applied a very thin coating of a less permeable polymer, SEBS-g-MA (maleic anhydride grafted styrene-ethylene-butylene-styrene). The SEBS-g-MA co-block polymer layer was applied in the final stage of manufacturing, using the same airbrush as the electrodes. For this purpose it was dissolved in Toluene at a concentration of 100mgmL^−1^. This allowed the structure to maintain its inflated state for a timespan on the order of 10 s of hours rather than minutes.

### Measurement

We used two methods for measuring actuation. First, optical tracking of markers placed on top of the actuators. For this we used TrackMate, an ImageJ plug-in (Tinevez et al., 2017). However this was limited in terms of resolution and sampling frequency. Our second method utilised a laser displacement meter (Keyence LC-2400A with an LC-2440 diffuse reflective sensor head) to track the displacement to an accuracy of 0.1 µm. The drawback for this method was that it could only measure displacement in one direction, and the laser range restricted actuator configurations.

For measuring the blocked force produced by the actuation of the ampulla, MIDA was placed such that the end effector region (see Figure 3) was in contact with a bolt head (area ^∼^76mm^2^) attached to a load cell (Interface SM-50N) whilst the ampulla was charged. The ampulla then had the voltage removed, pressing the end effector against the bolt.

## Results and Discussion

**FIGURE 10:**
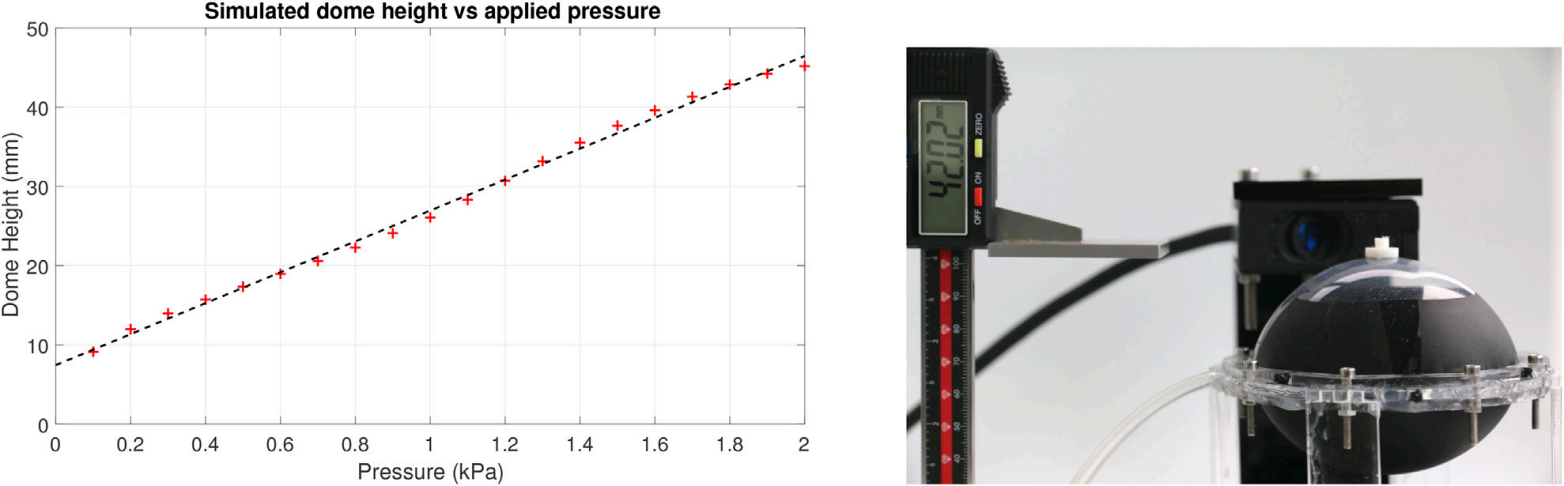
Left: FEM simulated dome height against internal pressure, linear fit. Right, experimental set-up showing the dome height measurement, tracking marker on the end effector, and the laser displacement sensor head in the rear.

Manual inflation was used for the purposes of characterising the MIDA actuators via a syringe attached to the air inlet tube. Our simulations showed a close linear relationship (*R* ^2^ = 0.997) between internal pressure and the height of the dome in the range 0.1–2 kPa, see Figure 10, this was used to set and maintain the desired pressure.

**FIGURE 11:**
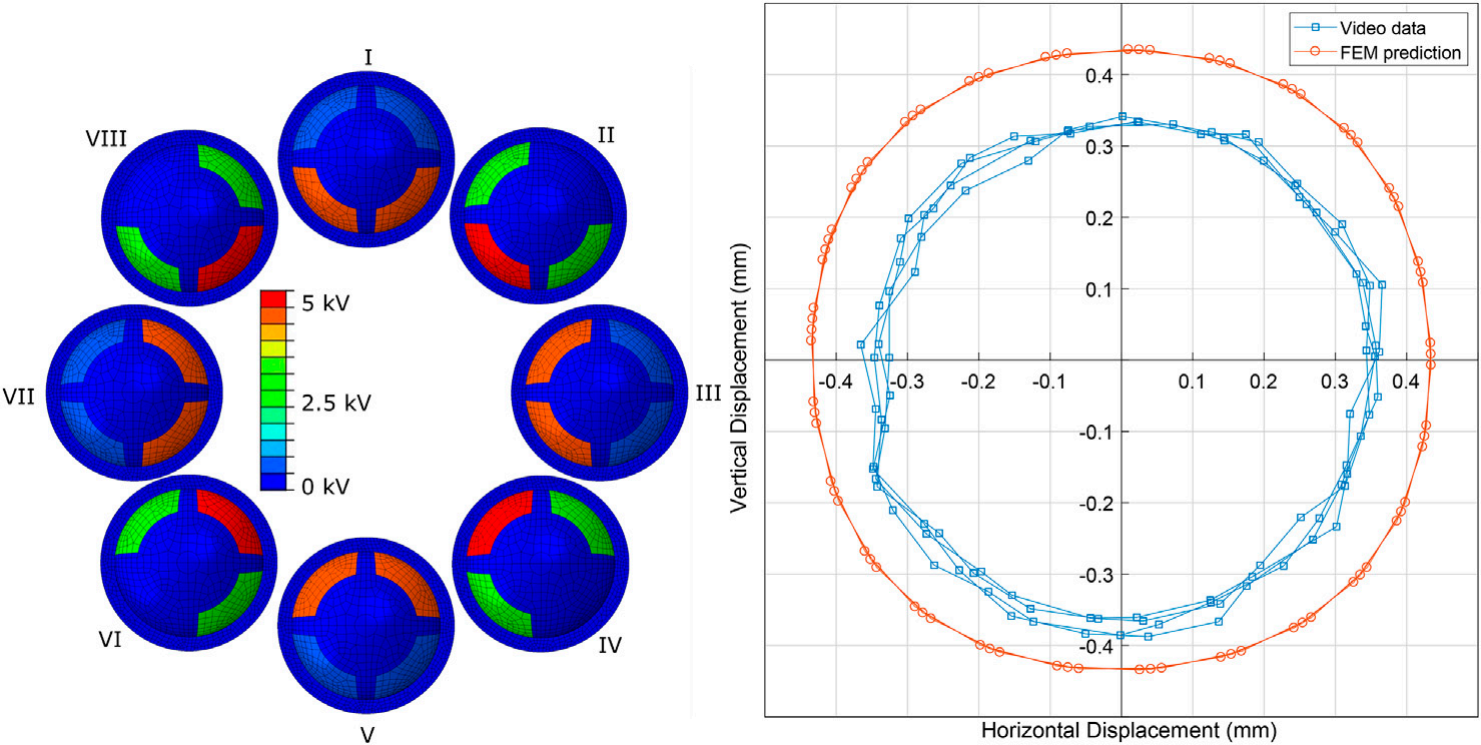
FEM Comparison. Left: MIDA FEM model showing the voltages applied to the segments during a ‘circular’ actuation, I - VII show the progression of the cycle. Right: A comparison of simulated (circular markers) versus experimental data (square markers) during actuation at 5 kV, with 90° phase staggering between adjacent electrodes. Data is shown for 3 cycles.

Figure 11 shows the measured tip displacement (using optical tracking) of a single sided MIDA prototype compared to the displacement predicted by the simulation under the same stimulation. The applied load used was a 1 Hz, 5 kV peak-to-peak amplitude sinusoidal wave with a 2.5 kV DC offset applied to all 4 electrodes with a 90° phase difference between adjacent electrodes. This caused the tip to move in a circular path on the X-Y plane (where Z is the normal to the membrane surface prior to inflation). The model overestimated the maximum displacement as it did not account for the additional stiffness produced by the electrode layers and additional SEBS layer, see Figure 8). These layers, though very thin (<10 *μ*m), had a significant impact on the actuation, demonstrating the impact even minor surface modifications have on DET performance.

**FIGURE 12:**
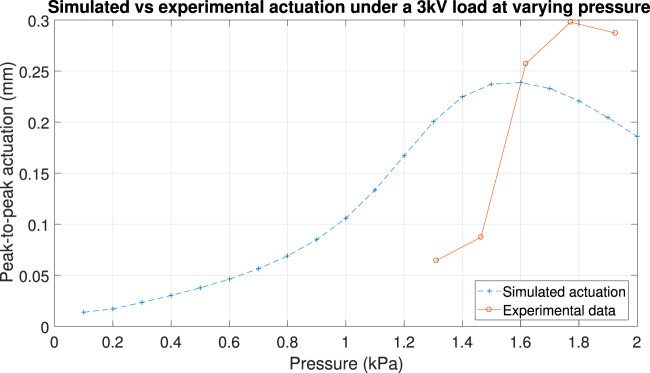
FEM Comparison 2. Simulated horizontal actuation (simultaneous actuation of two adjacent MIDA segments) under a 3 kV peak to peak sinusoidal load with 1.5 kV DC offset at varying pressure (blue) vs experimental actuation under the same conditions (orange) measure using the laser displacement sensor.

Using laser tracking, we measured the horizontal (in-plane) displacement of the tip of MIDA when two adjacent electrodes were actuated simultaneously with a 3 kV peak-to-peak amplitude sinusoidal wave with a 1.5 kV DC offset at increasing pressure. The amplitude of the actuation compared with our predicted displacement is shown in Figure 12. Although we see a similar trending curve it is not well aligned with the simulations. The optimum pressure was measured to be around 1.78 kPa rather than 1.6 kPa, a difference of around 10%, and the curve was notably taller and narrower than expected, with a much more pronounced increase in actuation and a maximum that was around 20% higher than expected. This further implied that the model used did not fully encompass the behaviour of the material, and required further refinement.

**FIGURE 13:**
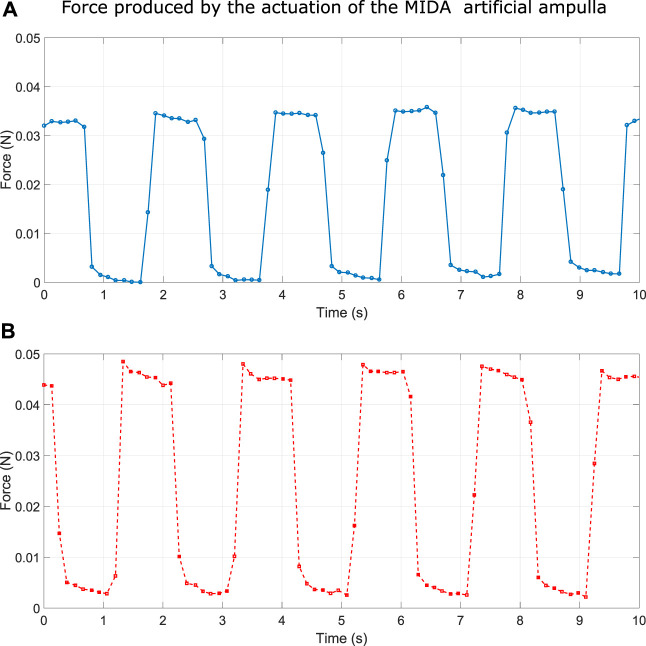
A: Force produced by MIDA ampulla actuated by a 3 kV square wave at 0.5 Hz. B: Force produced by MIDA ampulla actuated by a 5 kV square wave at 0.5 Hz.

The amount of force produced by MIDA was relatively low, and difficult to predict as it is a function several variables including; the level of inflation, and the amplitude and frequency of the applied voltage. Figure 13 shows the blocked force produced by the artificial ampulla at two different voltages, at the same frequency and constant inflation. The force produced was ^∼^35 mN for a 3 kV load and ^∼^45 mN at 5 kV.

Actuation of the ampulla and two adjacent segments using a square wave, out of phase by 90° produces the sequence seen in Figure 5B i–iv. Measured at 1.5 kPa, and using a 5 kV load, the tip of the end effector region followed a rectangular path which averaged 0.662 mm horizontally and 2.613 mm vertically. We can extrapolate this to a total operational volume being approximately a cylinder 1.324 mm in diameter and a height of 2.613 mm.

From our simulation we have extracted some measures of the forces produced by the side-to-side actuation of the MIDA segment of the actuator. This was accomplished by applying a translational constraint in the horizontal plane to a circular region of the inflated actuator 5.89 mm in diameter, and summing the reaction force in the direction of the wave. During a half-cycle, the average force produced was 9.9 mN with a peak of 16.5 mN.

We also used the simulation to predict the maximum displacement in the wave direction when the actuator moved freely, giving an answer of 1.27 mm (0.1 mm larger than the measured displacement). If we assume these values represent the edge cases of a work curve (maximum force with no displacement and vice versa) it would follow that the point halfway between them would represent a reasonable assumption of the maximum work. To that end, multiplying half of each value gave us 5.2 µJ of work, and thus a horizontal work density of 26.63Jm^−3^ given the volume of the membrane is 1.96 × 10^−7^m^3^.

In PILA, alternating large electrodes were actuated 180° out of phase, using a square wave with a constant offset voltage, such that they were always partially actuated. In our typical operation this was a 3 kV square wave with an offset of 2 kV, so that they switched between 2 and 5 kV. The offset helped ensure that the large electrodes maintained more distance between the object (or surface nearby) and the other portions of the actuator in order to avoid excess friction.

**FIGURE 14:**
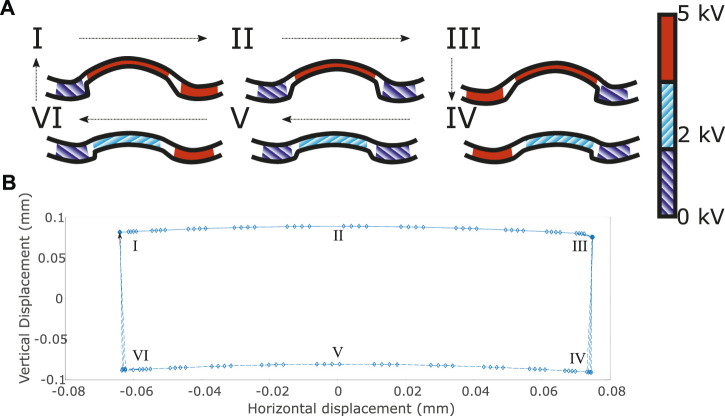
Path taken by the end point of each foot. **(A I–VI)** shows the actuation of the DEAs in a “foot” during the cycle. Red segments are actuated, blue segments are relaxed. **(B)**—FEM data of the expected tip displacement during four cycles using a 3 kV square wave with an offset of 2 kV applied to the “feet” and a 5 kV peak-to-peak sinusoidal wave with a 2.5 kV DC offset applied to the smaller electrodes. I-VI show the progression of the cycle on each diagram.

The smaller displacement electrodes were also alternately actuated 180° out of phase with each other, and they were also 90° out of phase with the large electrodes. Typically we used a 5 kV peak-to-peak sinusoidal wave with a 2.5 kV DC offset. This caused them to push the “feet” in one direction whilst the feet were raised and in the other direction when they were lowered, giving rise to the cyclic motion depicted in Figure 14.

These actuators have successfully demonstrated an ability to convey a long, lightweight object (a folded paper cuboid of dimensions 10 × 10 × 150 mm and mass of 0.25 g) along its length. The speed at which it did so was a function of the amplitude, and frequency of the applied loads, but it was also highly non-linear due to friction effects. Using the typical amplitudes described, earlier at 1 Hz, the load was carried at ^∼^0.06mms^−1^, whereas at 2 Hz it travelled at ^∼^0.96mms^−1^, and reached a maximum of ^∼^2mms^−1^ at 6 Hz.

## Conclusion

In this paper we have presented bio-inspired soft actuators mimicking the podia of starfish, using inflated dielectric elastomers as the primary component. We believe these actuators have great potential in the space sector, where many robotic systems are used, and a heavy emphasis is placed on reducing the mass of such.

Though the actuators demonstrated here, much like their biological inspiration, produce small displacements and low forces, this does not disqualify them for space operations. Given the latency of both sending commands and receiving data, the majority of space operations are performed at very slow speeds. This is compounded in low to zero gravity environments where stray forces have the potential to cause the spacecraft to drift away from its target/surface, potentially causing mission failure. This is also a strong argument for using electro-adhesive technology, as it can provide a stable adhesive force on almost any hard surface. Combining these, one application we envisage for the MIDA actuators is that of a gripper/manipulator for satellite-satellite interaction. Docking two spacecraft is a dangerous task, as the rigid mechanisms used must be precisely aligned and gently brought into contact or else risk substantial damage. This danger is compounded when the docking target is non-cooperative as is the case for defunct satellites or natural objects. Using soft grippers could allow for easier docking as they have inherent energy absorption properties and can deform to fit any surface. A claw with MIDA actuators on the tips would allow for soft gripping and manipulation of a target. A second application is as a locomotory system. Exploring the surface of objects with little gravity has proven to be a difficult task, as seen in the 2005 and 2015 minERVA and MINERVA-II rover missions (Yoshimitsu et al., 2006, 2016). Using electro-adhesion and the impact absorption of MIDA or PILA it should be possible to design a system capable of walking rather than hopping on these small bodies, allowing for more precise and safer movement. The surface gravity of the asteroid Ryugu, the target of the MINERVA rovers, varies from 0.110 to 0.145mms^−2^, roughly $\frac{1}{80\;000}$ times earth gravity (JAXA, 2018). This means that a single MIDA ampulla’s force of around 0.05 N (see Figure 13) would be capable of lifting a mass of over 450 kg.

In future we may wish to increase the force and/or displacements of our actuators, in order to increase their potential applications. Increasing the performance of DEAs has been the topic of a great deal of research in recent years, primarily through altering the materials used but also aspects of actuator construction: Reducing the thickness of the DEA layers reduces the required voltage to obtain a particular strain, due to the increased field density (see Eq. 2). By stacking multiple layers of DEAs you can dramatically increase displacement. This comes at a cost of lower tolerance for inhomogeneity or impurities in the dielectric layer, which can lead to actuator breakdown, and therefore an increased cost of fabrication and reduced reliability. Reducing the stiffness of a DEA will also increase actuation, either by reducing the Young’s modulus of the dielectric or making the electrodes more compliant. Most commonly, polymers are softened through the addition of plasticiser (Nguyen et al., 2009), or by reducing the amount of cross-linking agent/hardener used in their production (Zhang et al., 2005). Increasing the dielectric permittivity is another way to increase actuation, actuators with increased output have been developed by incorporating high dielectric particles such as titanium dioxide (Stoyanov et al., 2012) or barium titanate (Yang et al., 2016) into the dielectric layer or by blending elastomers with desirable properties (Carpi et al., 2008; Gallone et al., 2010). These approaches are complicated by the additional material requirements for usage in space (outgassing, operating temperature, and atomic oxygen resistance, etc), all new composites would need to be tested for space compatibility in addition to testing as a DEA. However, it may be possible to create an elastomer with improved actuation characteristics and with desirable environmental resistance properties, which would be ideal for our robots.

A potential concern is the high voltages required for both electro-adhesion and DEA actuation, however existing space technology, in particular gridded ion thrusters commonly use potential differences in the kilovolt range (Yang et al., 2020).

As mentioned previously, current pneumatic actuators are powered by large external compressors or stored compressed gas. Whilst these are potential methods for inflating our actuators, they are not feasible for small satellite based devices. The primary advantage of our actuators compared to standard pneumatics is that they only need to be inflated once, and the pressure maintained. The airtight layer requires improvement if these devices are to be used for extended periods, or the use of a continuous gas supply to counteract the losses. There have been studies that have developed elastomer materials capable of maintaining an effective gas barrier (Xiang et al., 2015; Ha et al., 2016), but their compatibility with our structures has not been assessed. Single-inflation actuators as a concept however opens different avenues for inflation/deployment methods.

Sublimating solids offer an attractive alternative. At low pressures some materials will vaporise, producing gas until they reach an equilibrium with their surroundings. This would allow us to include a small amount of solid material inside the actuator, which when launched would allow the device to passively inflate. The Echo 1 satellite used a combination of two different powders, benzoic acid to provide the initial inflation, and anthraquinone (which has a lower vapour pressure) to provide sustained inflation over the testing period of the satellite (1–2 weeks) (Clemmons, 1964). The amount of solid used determines the eventual pressure inside the inflatable, for example Echo 1 reached their desired skin stress (experimentally determined at a set temperature of 102 ◦C) with 4.54 kg of benzoic acid. Babuscia *et al.* calculate that for a 1 m parabolic reflector, 1 g of benzoic acid would suffice to maintain the internal pressure for over a year in low earth orbit (Babuscia et al., 2013). Preliminary experiments with benzoic acid in our laboratory have shown that it is also more than capable of inflating our much smaller structures. Using sublimating solids would allow our actuators to deploy and operate independently of an external inflation mechanism, making it much more similar to the starfish from which we take our inspiration and increasing the number of potential operating environments.

Space is a harsh environment for materials, with special considerations needing to be made for the radiation, extreme temperatures, vacuum, and other degrading factors. Whilst the membranes used in this study are not rated for space uses, silicones are very common in the space sector, and NASA maintains a large database of products which meet their outgassing standards (Knipple, 2008). Low earth orbit temperatures can fluctuate from around −65 to 125 ◦C, well within the acceptable limits of most silicones (for example, the elastosil membranes we used have a glass transition temperature of −126 ◦C, and a stated operating range of −45 to 200 ◦C). We are currently evaluating the impact of atomic oxygen exposure in low earth orbit on the mechanical properties of silicone elastomers, as well as developing and testing a method for shielding soft silicone structures from some of these factors.

## Data Availability

The raw data supporting the conclusion of this article will be made available by the authors, without undue reservation.
